# Tailoring Sensitivity and Selectivity with Nanoparticle-Functionalized ZnO Nanorods: The Impact of Metals on Sensing and Electrical Performance

**DOI:** 10.3390/s26113437

**Published:** 2026-05-29

**Authors:** Eray Tabak, Sadullah Öztürk, Arif Kösemen, Necmettin Kılınç, Zafer Ziya Öztürk

**Affiliations:** 1 Institute of Nanotechnology and Biotechnology, Istanbul University-Cerrahpasa, Istanbul 34450, Türkiye; eray.tabak@iuc.edu.tr (E.T.); arif.kosemen@iuc.edu.tr (A.K.); 2 Department of Physics, Inonu University, Malatya 44280, Türkiye; necmettin.kilinc@inonu.edu.tr; 3 Gebze Institute of Technology, Kocaeli 41400, Türkiye; zozturk@gtu.edu.tr

**Keywords:** ZnO, nanorods, nanoparticle, modification, sensor, hydrogen

## Abstract

In this study, metal (copper, nickel, cobalt, chromium)-decorated ZnO nanorods are successfully grown on glass substrates via a hydrothermal synthesis method to test their electrical and gas-sensing properties. SEM images revealed the formation of metal nanoparticles surrounding the ZnO nanorods. To confirm that these structures originated from the metal nanoparticles, EDX analysis was performed, and the presence of metal nanoparticles was validated. XRD analysis indicated that the crystal structure of the ZnO nanorods was hexagonal, and shifts in the (002) plane were observed due to metal nanoparticle doping. ZnO nanorods functionalized with metal nanoparticles were tested at 200 °C against various gases (hydrogen, ethanol, chloroform) and at different gas concentrations. The time-dependent variation in current was observed when ZnO nanorods functionalized with metal elements were exposed to hydrogen gas at test concentrations ranging from 1000 ppm to 5000 ppm at 200 °C. The results demonstrated a clear correlation between the rate of current change and hydrogen concentration, with higher concentrations resulting in faster responses. Additionally, the sensitivity of ZnO nanorods with decorated metal nanoparticles to ethanol and chloroform gases at concentrations ranging from 1000 ppm to 5000 ppm, as well as their sensor responses to different gases at 200 °C, were also measured.

## 1. Introduction

The increase in the amount of use of fossil fuels with the industrial revolution leads to the release of harmful gases into nature, negatively affecting the environment and human health. Gas sensors are used to detect life-threatening gases such as flammable, explosive, and suffocating gases that occur due to a wide variety of reasons (fossil fuel use and production processes, etc.). Gas sensors were first used to detect leaks in gas tanks or gas tankers by Oliver Johnson in 1926 [1].

In gas sensor applications and studies, semiconductor metal oxide materials have been used as a sensing layer in the detection and monitoring of pollutants, inflammable and explosive volatile organic compounds (VOCs), and toxic gases for many years due to maintain stable structures against the harmful physical effects of ambient temperature, humidity, pressure, and corrosion [2,3,4,5,6]. In chemical gas sensors, the success of semiconductor metal oxide materials in the field of gas sensors is not only limited to research but has also proven itself in commercial applications. Basically, a gas sensor consists of a sensing layer that can detect the gas in the environment, a transducer that can convert the interaction between the gas and the sensing layer into understandable physical quantities, and a user interface that can give a visual or auditory warning to the user by processing the signals coming from the converter. Tin oxide (SnO_2_), zinc oxide (ZnO), titanium dioxide (TiO_2_), and tungsten oxide (WO_3_) have attracted the attention of researchers with their high sensitivity and fast response time, especially in sensor studies, due to their wide electronic band gap and d-symmetry of their valence orbits [7]. The gas-sensing mechanism of metal oxides is realized by the charge exchange that occurs as a result of the reaction between the analyte gas molecules and surface trap levels composed of oxygen ions chemically binding to the sensitive material surface. Oxygen ions forming trap levels on the metal oxide surface exist in the forms of O^2−^ (<100 °C), O^−^ (between 100 °C and 300 °C), and O^2−^ (300 °C), depending on the surface temperature [8,9,10]. It has been demonstrated by scientific studies that doping and heterostructure applications have positive effects in order to increase the sensitivity and selectivity of semiconductor metal oxide material against tested gases [11]. In gas sensor studies, hetero-structured forms of metal oxides with metals or other materials offer advantages such as lowering the optimum operating temperature, increasing sensor stability, increasing resistance to moisture, reducing cross-sensitivity, and increasing selectivity, which is the weakest sensor parameter [12,13,14]. Yi et al. [15] obtained W-decorated NiO nanostructures and used them for NO_2_ detection. The pyramidal roughness formed on the surface of the W-decorated NiO facilitated interaction with target gas molecules, thereby increasing the gas sensing capacity. They achieved highly successful results, with a response time of two seconds and a recovery time of 60 s. Liu et al. [16] synthesized In_2_O_3_ modified with Pd nanoparticles. While the optimal operating temperature is typically around 200 °C, Pd loading reduced it to 135 °C. Karthik et al. [17] synthesized Cu-doped SnO_2_ nanomaterials, improving the sensor’s sensitivity to CO gas threefold and its response and recovery times twofold. Manikandan et al. [18] developed a Cr-doped Co_3_O_4_ gas sensor capable of detecting low concentrations of hydrogen sulfide (H_2_S) gas under near-room temperature conditions. These sensors could detect H_2_S gas at levels as low as 1 ppm in a short time and were proven to be stable and long-lasting even under conditions close to room temperature. Woo et al. investigated the gas-sensing properties of pristine ZnO nanowires and chromium oxide (Cr_2_O_3_)/ZnO nanowires obtained in different forms for understanding the positive effects of heterostructure use on sensitivity and selectivity. In particular, decorated ZnO nanowires showed better selectivity and sensitivity [19]. Navale et al. obtained CuO-ZnO nanorods using the thermal evaporation method and then observed an improvement in the NO_2_ sensing performance [20]. Chang et al. doped Pd nanoparticles on ZnO nanorods by the PVP-mediated photochemical deposition method. It was reported that the Pd nanoparticle-decorated ZnO nanorods responded approximately three times faster as a result of the Pd^2+^/Pd^0^ redox couple created by the palladium contribution, resulting in a higher electron-donating effect [21]. Yu et al. [22] obtained Pt nanoparticles and functionalized ZnO nanorods with the formation of Pt^0^ and PtO_x_ on the surface. PtO provides a p-n heterojunction with ZnO, which causes thickening of the transition layer. The thickening of the transition layer results in an increase in resistance. In this way, high selectivity gas sensing is realized. Despite the advantages of metal decoration, excessive doping can result in disadvantages such as reduced sensor performance and cross-sensitivity due to the inhibition of gas diffusion and electronic transport dispersion caused by dopant ion aggregation [23,24].

In this study, the changes in electrical properties and gas-sensing properties of ZnO nanorods decorated with metal nanoparticles produced by hydrothermal methods are investigated. The selectivity properties of the sensor device developed with the modification of metal nanoparticles differed. In addition, the gas-sensing mechanisms of the developed metal-decorated nanorods are explained.

## 2. Materials and Methods

### 2.1. Fabrication of Metal Oxide Nanorods

Basically, fabrication nanoparticle-decorated ZnO nanorods on the glass template was prepared in their steps as coating of a seed layer, growing of ZnO nanorods in a hydrothermal process, and nanoparticle modification. The ZnO seed layer was prepared by using a sol–gel process, which uses techniques for handling thin film on the surface.

For the production of the seed layer, the zinc acetate dihydrate salt (Zn(CH_3_COOH)_2_·2H_2_O) was prepared in ethanol with a concentration of 0.001 M and then stirred for two hours for a homogeneous solution with a magnetic stirrer. The acetate solution was coated with a cleaned glass substrate (ISOLAB microscope soda lime glass) by using a spin coater at 2500 rpm for 30 s and then dried at 130 °C for 5 min. This step was repeated five times, and finally, coated films were annealed at 300 °C in two hours for homogeneous structures. After annealing, the ZnO seed layer-coated substrates were stored in vacuum storage containers until the next experimental process.

For growing ZnO nanorods in the hydrothermal process, equimolar (0.1 M) zinc nitrate hexahydrate (Zn(NO_3_)_2_·6H_2_O) and hexamethylenetetramine (HMTA) solution were prepared in distilled water (18 Mohm·cm). Then seed layer-coated glass substrates were immersed into a sealable glass beaker and then aged at 90 °C for three hours to grow ZnO nanorods. Effects of seed layers on homogeneity of ZnO nanorods on the surfaces and dimensions of nanorods and the concentration of nitrate solution in the hydrothermal process were expressed in detail in our previous works [25,26,27].

To decorate the ZnO nanorods with copper, cobalt, chromium, and nickel metal nanoparticles, using acetate salts of each metal, solutions were prepared in ethanol with a concentration of 0.001 M. Prepared solutions were coated on the surfaces of the hydrothermally grown ZnO nanorods with the sol–gel process. In the sol–gel process, acetate solution was coated with a spin coater at 2500 rpm for 30 s, and the solution was dried at 130 °C for 5 min. These steps were repeated four times and finally annealed at 300 °C for 1 h. For morphological and structural characterization of the metal nanoparticle-decorated ZnO nanorods, X-ray diffractometer (XRD), scanning electron microscopy (SEM), and energy dispersive analysis systems were used.

### 2.2. Electrical Characterization and Gas Sensing Measurements

Gold interdigital transducer electrodes (IDE) were coated on the top surfaces of the metal nanoparticle-decorated ZnO nanorods with a thermal evaporation system (Leybold Univax450). The width of the metal electrodes in the IDE structure is 200 µm, with an inter-electrode spacing of 200 µm. DC electrical characterization was performed in two cycles between −1 V and +1 V with 0.05 V increments, starting from room temperature (25 °C) and increasing in 10 °C steps up to 200 °C. During the measurements, temperature was controlled using a Lakeshore 340A device, while electrical measurements were conducted using a Keithley 6517A electrometer/high resistance meter.

**Figure 1:**
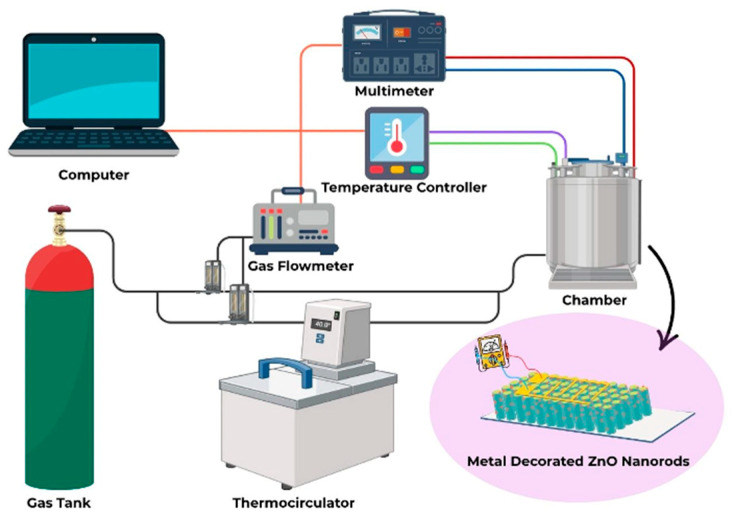
Schematically illustration of measurement set up.

To investigate gas sensing measurements of the metal nanoparticle-decorated ZnO nanorods, each sample was placed into the measurement cell. Among the gas sensing measurements, ultra-pure dry air was used as a carrier gas. Before gas sensing measurements, the measurement cell was purged with ultra-pure dry air for 1 h for cleaning contaminants on the surface of the fabricated sensor devices. The air flow rate in the measurement cell was set as 200 mL/min and controlled by mass flow controllers. When the sensor signal reached the steady state level (called as baseline), desired analyte and its lowest test concentration were purged into the measurement cell for approximately 20 min, and then dry air was purged into the measurement cell for cleaning sensor surfaces. These steps were repeated for other test analytes and the test concentrations. A schematic illustration of the measurement setup was given in Figure 1.

## 3. Results and Discussion

### 3.1. Structural Characterization

The production of ZnO nanorods by the hydrothermal method was carried out by the sol–gel method on a glass substrate coated with ZnO thin film (as a seed layer). It has been understood because of experimental studies that the seed layer acts as a trigger and guide in the growth of ZnO nanorods [28]. The pH value of zinc nitrate solution in water is around 5. As the solution temperature increases (the hydrothermal process realization temperature was 90 °C), the pH of the solution decreases and becomes acidic. The shift in the pH balance to acidity brings along the etching process and prevents the formation of nanostructures. On the other hand, it has been observed by studies that HMTA, which is added into the solution in equal concentration, increases the pH value of the solution to around 10, since it continuously provides hydroxyl ions (OH^−^) into the solution [29]. However, the hydroxyl ions they provide into the solution react with the Zn^2+^ ions provided by the zinc nitrate salt, resulting in ZnO. Chemical reactions that take place during the hydrothermal process were given Equations (1)–(4) [30,31].

(1) $${\left({CH}_{2}\right)}_{6}6N_{4}+6H_{2}O\to 6HCHO+4NH_{3}$$

(2) $${NH}_{3}+H_{2}O\to {{NH}_{4}}^{+}+{OH}^{-}$$

(3) $${Zn}^{2+}+2{OH}^{-}\to Zn(OH{)}_{2}$$

(4) $$Zn(OH{)}_{2}\to ZnO+H_{2}O$$

Since the Zn(OH)_2_ molecule, which is released as a result of the combination of Zn^2+^ ions and OH^−^ ions, is more unstable than the ZnO molecule, it dissolves in the solution and turns into a ZnO molecule. As a result of the chemical reactions that take place, the water molecule and ZnO molecule are revealed in the solution in the medium, and the Zn and O atomic planes, which are arranged sequentially along the [0001] and $\left[000\underline{1}\right]$ planes form a tetragonal arrangement in the crystal structure. Since Zn and O atoms are in different electrical charges in the tetragonal arrangement, it causes local dipole moments to form in the crystal structure. The direction of the dipole moment formed is in the same direction as the ZnO polar crystal cross-section planes. The dipole moment reverses with the deposited atomic layer, resulting in the formation of planes consisting of Zn and O atoms in an ordered state [31].

**Figure 2:**
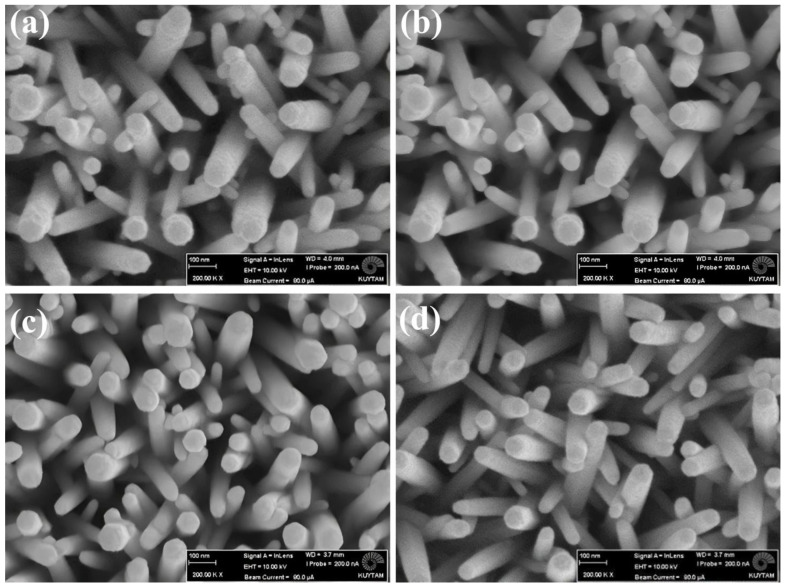
SEM images different metal nanoparticle-decorated ZnO nanorods on top view (**a**) Co, (**b**) Cu, (**c**) Cr ve, (**d**) Ni.

Metal nanoparticles were formed on ZnO nanorods by the sol–gel method, which is a solution-based growth technique. With the final state to be reached, it is possible to increase the catalytic effects and surface area of the point metal particles and also to improve the gas detection properties. Metals formed on ZnO nanorods are cobalt (Co), copper (Cu), chromium (Cr), and nickel (Ni). The surface characterizations of the samples produced after the synthesis processes were carried out with the SEM device. SEM images of heterostructure samples obtained with different metal nanoparticles were given in Figure 2.

**Figure 3:**
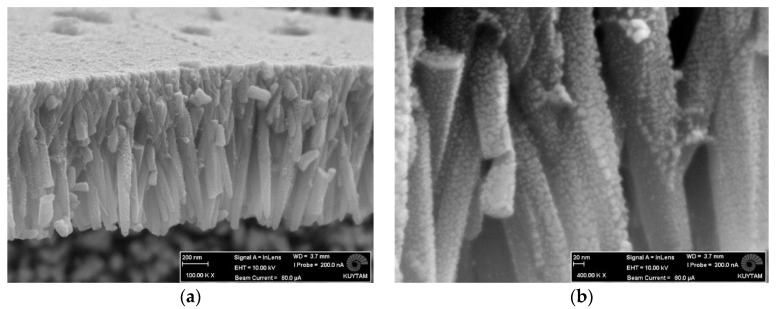
Cross-sectional view of Cr metal nanoparticle-decorated with ZnO nanorods (**a**) low magnified and (**b**) high magnified SEM images.

As evident from the SEM images, the ZnO nanorods retain their structural integrity after the metal decoration process, with no observable morphological deformation when compared to those presented in our previous studies [28]. It was determined that metal nanoparticles were formed around the nanorods by examining the produced samples at higher resolution. SEM image of Cr nanoparticles on ZnO nanorods is given in Figure 3. As can be seen from Figure 3, when the lateral surfaces of ZnO nanorods are examined at high magnification, it is seen that there is a particulate structure. This situation is not only limited to the ZnO nanorod sample equipped with Cr metal nanoparticles but also observed in other samples.

**Figure 4:**
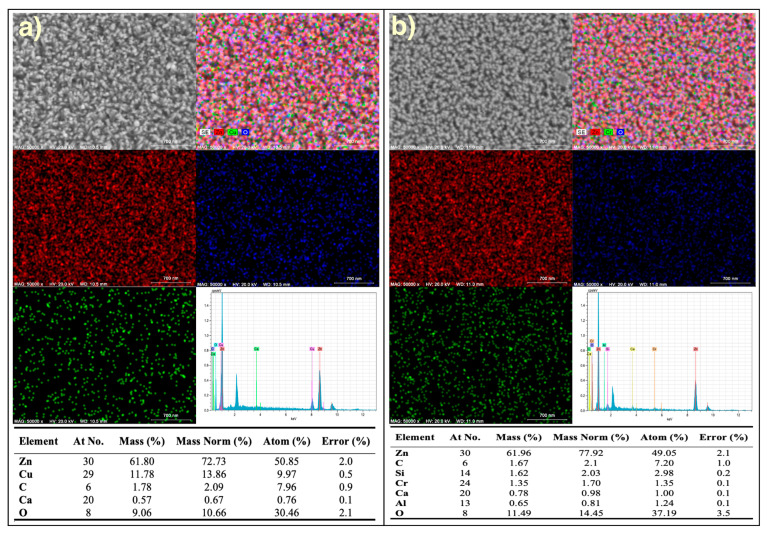
EDX analysis of metal nanoparticle decorated ZnO nanorods decorated with (**a**) Cu and (**b**) Cr.

The elemental composition and spatial distribution of the metal nanoparticle–modified ZnO nanorods were examined by using energy-dispersive X-ray spectroscopy (EDX) expanded with elemental mapping technique, as presented in Figure 4. For both samples (a) and (b), the top left images show the secondary electron micrographs of the modified ZnO nanorods, while the top right images present the corresponding overlay of all elemental map results, and different colors were used for allocating elements (red for Zn, blue for O, and green for modification elements). The even coloration across the mapped regions presents a relatively homogeneous surface coverage of each element. The EDX spectrum of both samples indicates characteristic peaks for Zn, O, and modification elements (Cu for Figure 4a and Cr for Figure 4b, respectively), verifying the intended modification of the ZnO nanorods with metal nanoparticles. Quantitative analysis results are listed in the tables below each figure. The mapping results, in conjunction with the EDX spectra, confirm that the modified metal nanoparticles are well-dispersed over the ZnO nanorod surfaces rather than forming large agglomerates. These situations are essential for maintaining uniform surface properties and enhancing the functional performance of the nanostructures. According to EDX analysis and elemental mapping results, could be commented that successful incorporation of the metallic component into the ZnO matrix while retaining the structural integrity of the nanorods.

**Figure 5:**
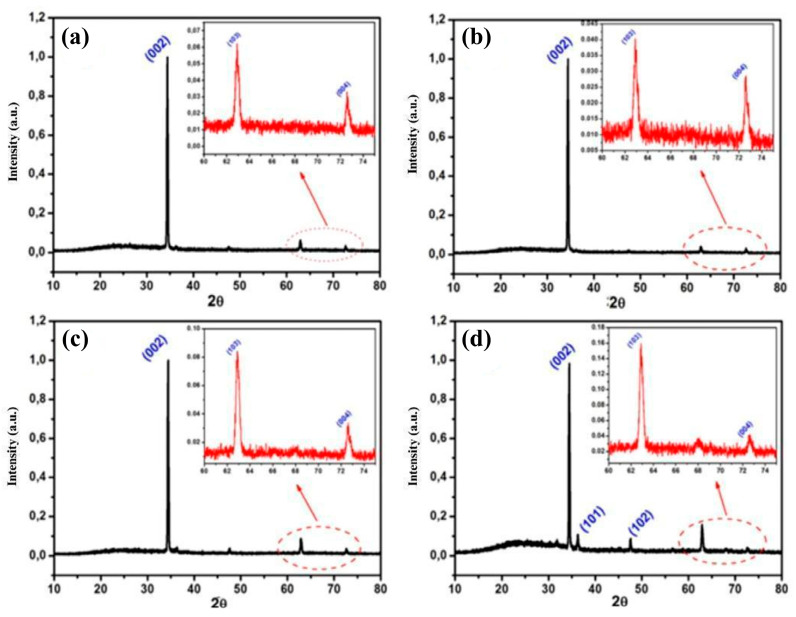
XRD graphs of heterostructured ZnO nanorods decorated with different metals (**a**) Co, (**b**) Cu, (**c**) Cr, (**d**) Ni.

XRD analysis was carried out to examine the effect of metal nanoparticles on the structural properties of ZnO nanorods. XRD graphs of metal nanoparticle ZnO nanorods are given in Figure 5. It is understood that the samples produced in the XRD analysis graphics have exactly similar crystal planes with the hexagonal ZnO nanorods [25,32].

**Figure 6:**
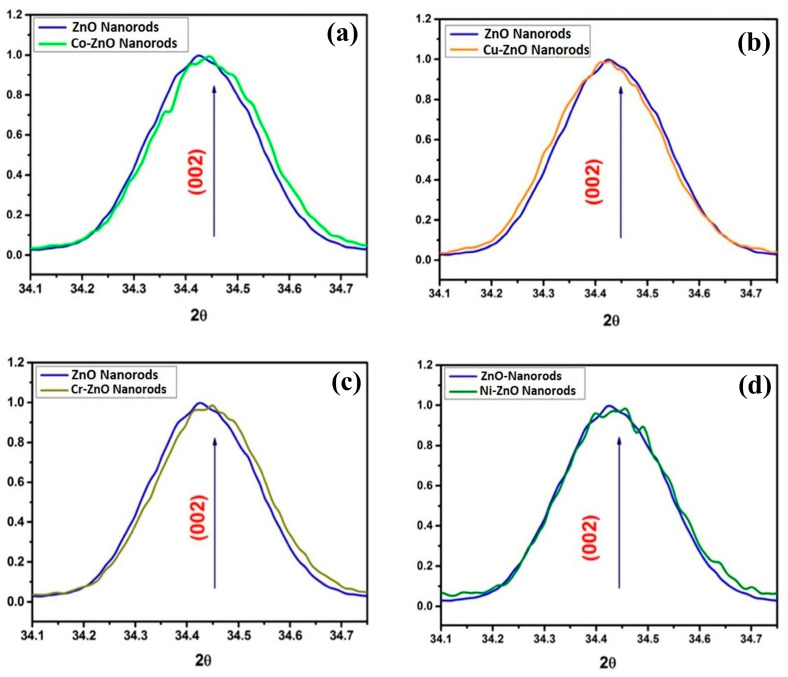
Position of (002) peaks in XRD graphs of heterostructured ZnO nanorods decorated with different metals (**a**) Co, (**b**) Cu, (**c**) Cr, (**d**) Ni.

On the other hand, it is seen that there are differences in the reflection intensity values of the peaks belonging to the (103) and (004) indexed crystal planes. This situation has emerged with the literature review that changes depending on the amount of material deposited on the ZnO nanorods [33]. In similar studies, in heterostructured samples, and in studies obtained by doping, it was determined that shifts occurred at the angles where the diffraction peaks in the XRD graph were observed [19,34]. The XRD curves of the diffraction peaks observed at an angle of 34° are given in Figure 6. With the enrichment with metal nanoparticles, the diffraction peak of the (002) index crystal plane of ZnO shifted to the right or left. It has been understood by literature studies that the atomic sizes of the metal ions formed on ZnO are caused by the difference in the atomic sizes of the Zn atom [34]. The amount of Fe_2_O_3_ is too small; the XRD patterns of the two samples are almost unchanged from pure ZnO, and no obvious Fe_2_O_3_ peaks can be detected. A similar phenomenon is also reported in other literature studies [35].

**Figure 7:**
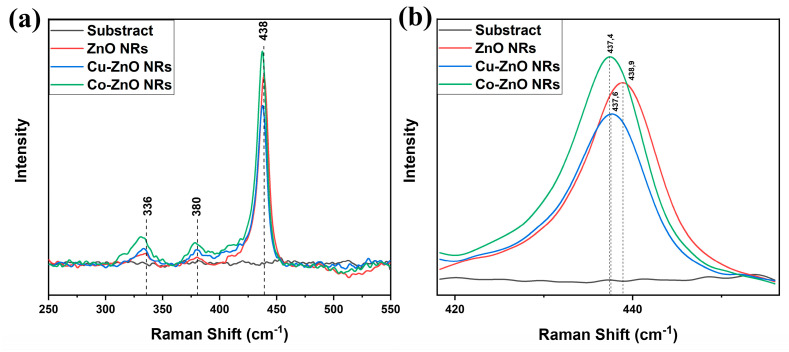
(**a**) Raman spectra of undoped ZnO and doped ZnO, (**b**) The spectrum of the undoped and doped ZnO at 438 cm^−1^.

The Raman spectrum was measured in the 100–800 cm^−1^ range using a 532 nm laser. Figure 7 shows the Raman spectra of subtract, decorated (Cu and Co), and bare ZnO nanorods. According to Figure 7a, dominant peaks at 334, 380, and 438 cm^−1^ appear in all samples. These peaks are characteristic optical modes of hexagonal wurtzite ZnO and can be attributed to the A1(TO), E1(TO), and E2H modes of ZnO, respectively [36,37,38,39]. Figure 7b shows that doping ZnO nanorods with Cu and Co causes a specific shift in the Raman active mode (438 cm^−1^). The peak at 438 cm^−1^ in pure ZnO shifts to 437 cm^−1^ in the presence of dopants. According to J. Ridwan et al. (2022), this shift towards higher wavenumbers in the Raman spectrum peaks can be attributed to strain in the crystal lattice [40]. The chemical bond length in the ZnO lattice has been altered by Cu and Co [40]. The shifts observed in the XRD patterns and the shift in the Raman active mode support the change in the lattice structure of the sample. These results indicate that Cu and Co have been successfully incorporated into ZnO NRs [41,42]. However, due to the very low doping concentration, only a slight shift was observed, and no Cu- or Co-derived peaks were detected.

**Figure 8:**
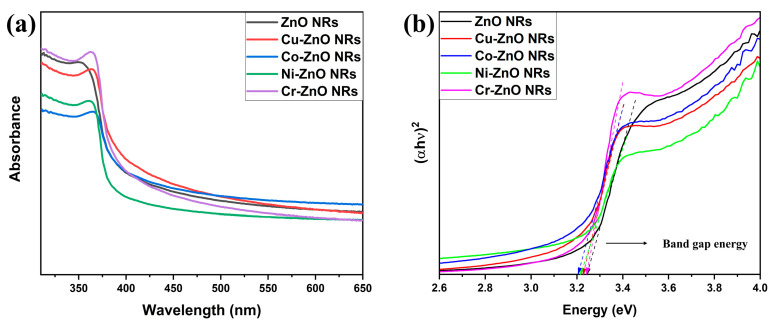
(**a**) UV-vis absorption spectra for all ZnO NRs, (**b**) bandgap energy of all samples.

UV-vis absorption spectroscopy was applied in the 200–800 nm range to investigate the optical behavior of pure and metal-decorated ZnO NRs. According to Figure 8a, pure ZnO showed a strong peak at approximately 350 nm. Compared to previous studies, this indicates that ZnO is UV-active and corresponds to an intrinsic band transition [43,44,45]. Compared to pure ZnO, differences in the absorption edge are noticeable in metal-decorated ZnO NRs. Metal-decorated NRs exhibited a red-shifting behavior. According to Qamar et al. (2022), this tendency may originate from hybridization between Zn atoms and metal ions [46]. In this case, the metal doping causes band narrowing in the sample, leading to a decrease in the band gap energy. The band gap energy of metal-decorated and pure ZnO was calculated using the Tauc equation in this Equation (5), and the band gap energies of ZnO NRs are shown in Figure 8b. Accordingly, the band gap energies of pure and metal-decorated ZnO NRs (Cu, Co, Ni, Cr) are 3.25, 3.22, 3.20, 3.21, and 3.24 eV, respectively. As can be seen here, band narrowing resulted in a decrease in band gap energies with low metal concentration. These results are consistent with those reported in the literature. The reduction in *E_g_* is attributed to sp-d exchange between ZnO band electrons and metal ion d electrons [47,48].

(5) $$ahv={A(hv-E_{g})}^{n}\;$$

where *α* is the absorption coefficient, *hv* represents the photon energy, *E_g_* is the band gap, *A* is a proportionality constant, and *n* denotes the transition type. Since ZnO exhibits a direct band gap transition, *n* was taken as 2.

### 3.2. Electrical Properties

**Figure 9:**
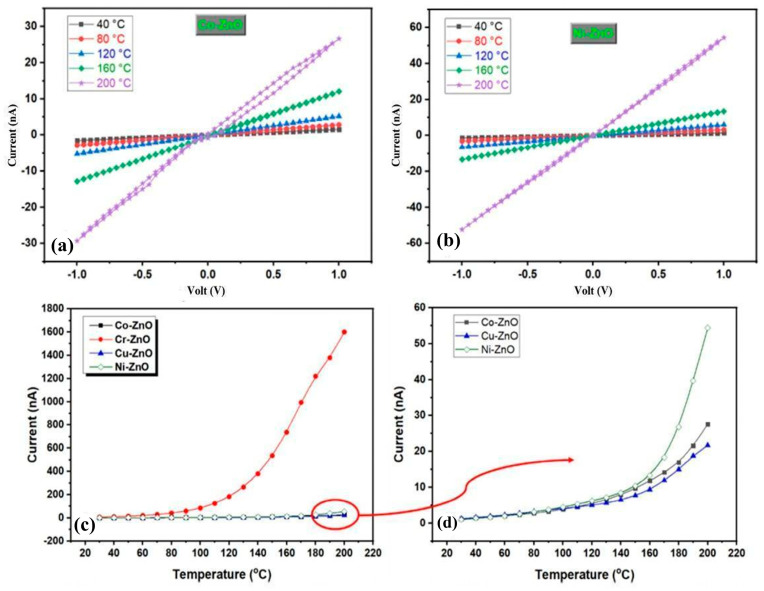
(**a**) The I-V characteristics of Co decorated and (**b**) Ni decorated (**c**,**d**) ZnO nanorods at different temperatures. The current that measured by applying 1 V as a function of temperature for metal decorated ZnO nanorods.

DC electrical characterization of all metal-decorated ZnO nanorods was evaluated with I-V measurements at different temperatures from RT to 200 °C. Figure 9a,b show I-V graphs for Co- and Ni-decorated ZnO nanorods at the indicated temperatures. A linear relationship between current and voltage in the temperature and voltage intervals, as seen in Figure 9a,b. Similar behavior is observed for Cr- and Cu-decorated ZnO nanorods. In general, there is a Schottky contact between ZnO and Au metal contact [49,50]. On the contrary, some studies observe Ohmic behavior between Au and ZnO. Gu et al. fabricated Au on an n-type ZnO 0001 single crystal substrate, and they obtained Ohmic behavior for the Au/ZnO contacts fabricated on substrates without H_2_O_2_ pretreatment in the voltage interval of –2 V to +2 V [51]. Mosbacker et al. investigated the behavior of metal/ZnO contacts formed with different metals, and they found that defects generated at higher temperatures and/or with higher initial defect densities for all interfaces produced Ohmic contacts [52]. Rana and Kim observed Ohmic behavior for the Au/ZnO nanorods/Au sandwich structure that was prepared on a flexible substrate via the aqueous chemical growth (ACG) method, and they explained this behavior with high surface and subsurface level donor defect-centric Schottky barrier pinning at the Au–ZnO interface [53]. Lin et al. prepared Au contacts on sol–gel ZnO films, and the obtained Ohmic behavior was explained with the existence of native defects associated with oxygen and zinc [54]. In a previous study, we performed I-V measurements on bare ZnO nanorods in the same electrode structure and observed Schottky behavior in the applied voltage range of −50 V to +50 V [27]. In our case, for metal-decorated ZnO nanorods, we did not see Schottky behavior in the measured temperature and voltage intervals. As a result, the Ohmic behavior we obtained in the Au/nanoparticle-decorated ZnO nanorod structure can be explained by defects, heterostructures formed by nanoparticles on the nanorods, or the applied low voltage range. Figure 9c gives the temperature-dependent current at 1 V of applied voltage for all metal-decorated ZnO nanorods. The current of metal-decorated ZnO nanorods increased with temperature, and this behavior could be related to the fact that the samples are semiconductors. The order of current values of metal-decorated ZnO nanorods at the temperature of 200 °C from largest to smallest is Cr, Ni, Co, and Cu.

**Figure 10:**
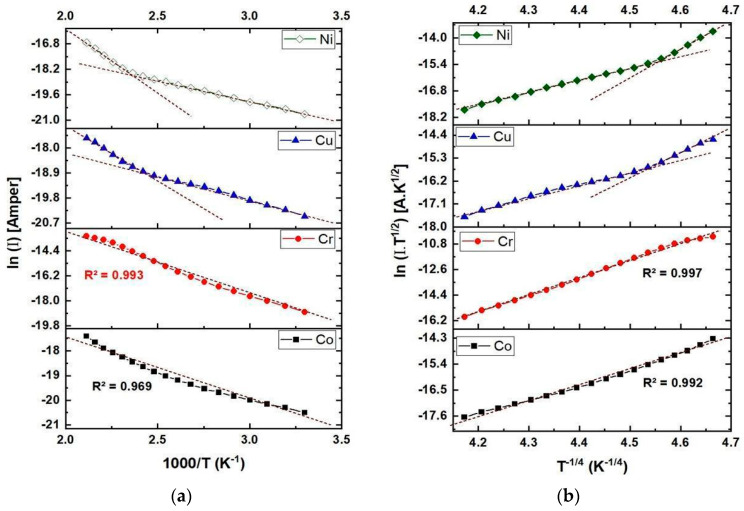
Temperature dependency of metal nanoparticle decorated ZnO nanorods.

In order to elucidate the DC conduction mechanism, temperature-dependent I-V graphs were used. The dc conductivity value was not calculated because the nanorods between the two electrodes are not continuous; they are perpendicular to the surface, and even if the nanorod bases touch each other, there is a distance between the tip regions of the nanorods. Instead, the current value expected to be directly proportional to the conductivity of the material was considered. Therefore, instead of dc conductivity, the current value is taken, and the dependence of the current on the reciprocal absolute temperature (1/*T*) could be defined using the following Equation (6):

(6) $$I=I_{0}exp\left(\frac{{-E}_{a}}{kT}\right)$$

where *I*_0_ is the constant of proportionality, *E_a_* is the activation energy, *k* is the Boltzmann’s constant, and *T* is the temperature. In Figure 10a, the ln (*I*)−1000/*T* graph is given for all metal-decorated ZnO nanorods. A linear behavior with two distinct regions was observed for Ni- and Cu-decorated ZnO nanorods related to two activation energies. For Cr-decorated ZnO nanorods, a linear behavior was observed between ln (*I*)−1000/*T*, and the coefficient of determination (or linearity factor, R^2^) value was obtained as 0.993. Therefore, this situation can be explained by thermal activation. On the other hand, the coefficient of determination (R^2^) value obtained from the ln (*I*)−1000/*T* graph for Co-decorated ZnO nanorods is 0.969. The calculated activation energies of all metal-decorated ZnO are in the range of 0.16–0.63 eV, as given in Table 1. Especially due to the low coefficient of determination (R^2^) value for Co-decorated ZnO nanorods, the suitability of the “*Variable Range Hopping* (VRH) model” proposed by Mott for all-metal-decorated ZnO nanorods should be taken into consideration. The VRH model for semiconductors is predicted by the following Equation (7) [55,56].

(7) $$\sigma_{dc}\sqrt{T}=\sigma_{0h}exp\;{\left(-\left(\frac{T_{0}}{T}\right)\right)}^{\frac{1}{4}}$$

where *σ*_0*h*_ and *T*_0_ are given by the following Equations (8) and (9).

(8) $$\sigma_{0h}=3e^{2}\upsilon_{ph}{\left(\frac{N\left(E_{f}\right)}{8\pi \alpha k}\right)}^{\frac{1}{2}}$$

(9) $$T_{0}=\frac{\lambda a^{3}}{KN\left(E_{F}\right)}$$

where *ν_ph_* is the phonon frequency at Debye temperature, *α* is the inverse localization length of the wave function for the localized state, *N*(*E_f_*) is the localized state density for electrons at the Fermi level, and *λ* = 16. In general, the VRH model explains the charge transport mechanism between localized energy states that could be related to distorted bond angles, defect centers, and impurities. In order to test whether the electrical conduction mechanism of all metal-decorated ZnO nanorods is suitable for the VRH model, the plot of ln (*I*·*T*^1/2^) versus *T*^−1/4^ is plotted and shown in Figure 10b. A linear behavior with two distinct regions is observed for Ni- and Cu-decorated ZnO nanorods, similar to the thermal activation model. Also, the coefficient of determination (R^2^) value increased slightly for Cr-decorated ZnO nanorods. However, the linearity between ln (*I*·*T*^1/2^) and *T*^−1/4^ was very good, and the coefficient of determination (R^2^) value is greater than 0.99 for Co decorated ZnO nanorods. In this case, the VRH model is more suitable for Co-decorated ZnO nanorods, while the thermal activation model can be suggested for other metal-decorated ZnO nanorods.

The same result was also proved by Kumar et al. [57]. In order to see the effect of temperature on electrical conductivity, ZnO and Co-doped ZnO thin films were deposited on sapphire substrate by ultrasonically assisted chemical vapor deposition technique. While the dominance of thermally activated band conduction was observed in the high-temperature region, hopping conduction was found to be dominant in the low-temperature region. Dar et al. [58] have shown that in Ni doped ZnO thin films, the Mott VRH conduction process is dominant at very low temperatures, while thermal activation behavior is dominant at relatively higher temperatures.

### 3.3. Gas Sensing Properties

**Figure 11:**
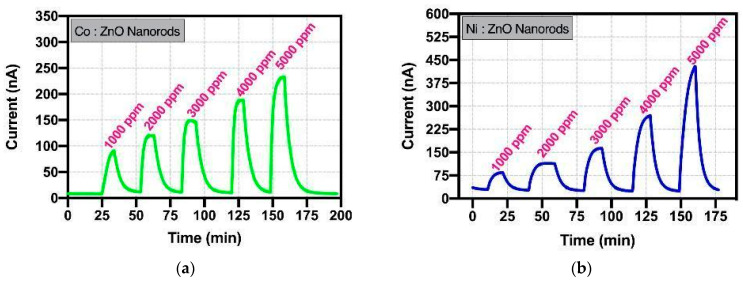
Hydrogen sensing responses of (**a**) Co and (**b**) Ni metal nanoparticles decorated ZnO nanorods at 200 °C.

ZnO nanorods functionalized with metal nanoparticles were tested against two different gases and different concentrations at 200 °C. In Figure 11, the graph of variation in the current versus time was given by exposing the ZnO nanorods functionalized with Ni and Co to hydrogen gas at test concentrations from 1000 ppm to 5000 ppm at 200 °C.

**Figure 12:**
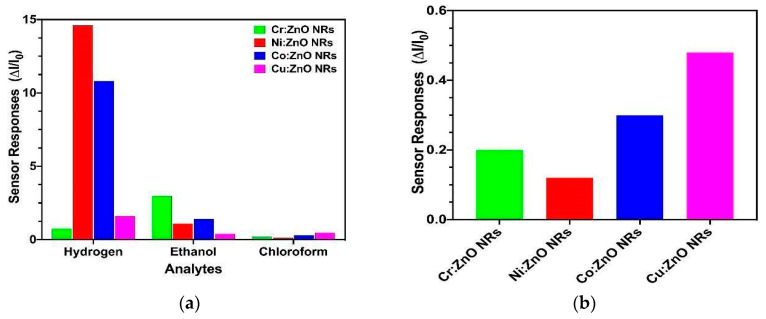
Sensitivity values of metal nanoparticle decorated ZnO nanorods for (**a**) all tested analytes (**b**) only chloroform.

The sensor responses were calculated by using formula as below Equation (10).

(10) $$Sensor\;Response=\frac{∆I}{I_{air}}=\frac{I_{gas}-I_{air}}{I_{air}}$$

here *I_gas_* and *I_air_* are the current values of sensor devices under indicated gas and air conditions, respectively. It can be clearly seen that with the increase of hydrogen concentration, the amount of change in current increases more rapidly. In addition, the sensitivity values of ZnO nanorods decorated with metal nanoparticles to ethanol and chloroform gases at concentrations increasing from 1000 ppm to 5000 ppm at 200 °C temperature and the sensor responses of different gases are given in Figure 12. In our previous work, bare ZnO nanorods showed sensor responses as 1 [27], but Ni nanoparticle-decorated nanorods showed sensor responses of approximately 14.83 at the same testing conditions (for exposure to 1000 ppm H_2_ at 200 °C).

It is clearly seen that the gas sensing responses change with the change in metal nanoparticles on the surface of the metal oxide nanorods. The difference in sensor responses is very important in terms of detecting the gases in the environment in a distinguishable way. The interaction between metal atoms and metal oxide with gases is explained in two different ways. The first is called electronic sensitization, while the second is called chemical sensitization [59]. In chemical sensitization, metal nanoparticles on the surface of the metal oxide nanostructure cause the formation of barrier layers in small regions. The width of the barrier depends on the relative positions of the work functions of the metal atom and the metal oxide [60]. For this reason, the barrier width is different for each metal and affects the amount of current flowing and therefore the amount of carrier load. Since the charge concentration has a key role during the detection of the gas, the amount (concentration) of the charges arising or lost because of the interaction of the gas species with the metal oxide will differ, and the currents observed during the gas tests will be different. The difference in current reveals an important result in terms of improving the selectivity parameter, which is an important problem in the production of gas sensors.

The fact that the semiconductor metal oxide in sensor applications has a wide band gap and low activation energy is a positive situation in terms of sensor properties. Since the forbidden band gap is high, the change in conductivity, which is a result of the electron exchange that occurs as a result of the interaction of the surface with the gas, is noticeable [61,62]. Alev et al. [63] synthesized Cu-doped ZnO nanorods using the electrochemical deposition method and demonstrated their effectiveness in detecting harmful gases (isopropyl alcohol, dichlorobenzene, ethyl acetate, toluene, xylene, H_2_S, HCN, and NO_2_). Their study revealed that Cu doping increased oxygen vacancies on the ZnO nanorod surface, transforming ZnO into a Lewis base and enhancing adsorption sites. This modification facilitated greater electron release, improving the sensitivity of gas sensing. The most efficient results were obtained with 1% Cu-doped ZnO nanorods. Yuan et al. [60] reported that the Co-modified ZnO nanorods they developed improved the gas-sensing properties for n-propanol. They concluded that Co doping not only provided numerous adsorption-active sites for oxygen molecules and n-propanol but also optimized the gas-sensing performance by reducing the resistance of ZnO through the reintroduction of additional free electrons. The enhanced gas-sensing properties of the Co@ZnO nanorod sensor toward n-propanol were attributed to the catalytic oxidation of n-propanol, the unique nanorod morphology, and the shift in ZnO’s Fermi level induced by Co doping.

Table 2 summarizes the metal oxide-based gas sensor studies previously reported in the literature. In addition, the table also includes literature findings on metal oxides functionalized with noble and non-noble metal nanoparticles in different morphologies (e.g., nanosheet (NS), nanowires (NWs), nanorods (NRs), nanoparticles (NPs), and also thin films (TF)), thus demonstrating the effect of morphology and functionalization type on sensor performance.

However, the general characteristic of non-transition element metal oxides is that the valence orbitals of metal atoms have s- and p-symmetry, allowing only one oxidation level. The low level of oxidation increases the difference between the allowed energy levels to which electrons can pass, and therefore, very high energies are required to remove or lose an electron from the metal atom bonded with oxygen. The reason why oxidizability levels are high in transition element metal oxide materials with d-symmetry is due to the fact that the valence orbital of metal atoms in transition element metal oxide materials is partially filled with electrons [2,10]. The low energy difference causes oxidation levels to occur at low and high energies when metal oxide materials with transition elements are present. The increase in permissible levels of oxidation allows the creation of defects in metal oxide materials with transition elements, and the electronic structure of which can be changed easily [2,10]. The most suitable metal oxides for sensor applications should have a d^0^ (i.e., TiO_2_, V_2_O_5_, MoO_3_, WO_3_, Nb_2_O_5_, ZrO_2_, and HfO_2_ ) or d^10^ (i.e., ZnO, CdO, HgO, Cu_2_O, Ag_2_O, and Au_2_O) electronic arrangement and a band gap of 3 to 4 eV. Studies have revealed that the electron configurations in the d orbital of the metal atom forming the metal oxide are more effective in the form of d^3^ and d^6−8^ [3]. It has been observed that the catalytic properties are surprisingly reduced with the addition of metal oxide materials with a d^5^ electronic array added to the sensing material. It has been shown that it is more convenient to use metal oxide materials with d^0^ and d^10^ electronic arrays as the main sensing layer instead of using them to increase the catalytic properties [11]. Basak et al. [70] reported that Co-doping in ZnO nanorods synthesized via the hydrothermal method enhances surface defects, thereby influencing their chemical and electronic properties. Upon exposure to H_2_ gas, the adsorbed oxygen species are reduced by H_2_, releasing a significant number of trapped electrons and increasing conductivity. Co-doping was found to create abundant oxygen vacancies associated with donors, providing more adsorption sites for O_2_ molecules and leading to a thicker depletion layer with higher barrier height. This results in surface resistance being significantly higher than bulk resistance, and conductivity near the surface is considerably lower compared to pure ZnO. ZnO nanorods doped with 8% Co exhibited approximately three times higher gas response than undoped nanorods.

Modification of the nanorod surface of the metal oxide chosen as the sensing element with nanoparticles of functionalizing elements (both metallic and oxide forms) improves the catalytic activity of the surface in the detection of target gas molecules. Functionalizing elements not only accelerate the adsorption–desorption kinetics of gas molecules by creating additional chemisorption centers on the surface but also support the improvement of sensor sensitivity by facilitating the separation of intermediate species formed during surface reactions. In addition, the band alignment and band bending effects occurring in the heterojunction regions between the metal oxide nanorod, which is the basic sensor material, and the functionalizing element cause the redistribution of electron carriers, causing the electron depletion layer in the junction region to narrow. This allows charge transfers resulting from chemical reactions between target gas molecules and the sensor layer to be met with lower energy requirements. In this context, functionalized metal oxide nanostructures not only increase catalytic activity but also improve gas sensing mechanisms through electronic modulation provided by heterojunction regions.

## 4. Conclusions

The hydrothermal synthesis of ZnO nanorods decorated with metals (copper, nickel, cobalt, chromium) was successfully achieved on glass substrates coated with ZnO thin films (seed layers) via the sol–gel method. SEM analysis revealed the formation of metal nanoparticles surrounding the ZnO nanorods. To determine whether the particle structures around the ZnO nanorods originated from solution-induced erosion or from metal nanoparticles, EDX analysis was conducted. The EDX results confirmed that material accumulation on the side surfaces of the nanorods was due to the metal nanoparticle modification process. XRD analysis demonstrated that the produced samples shared identical crystal planes with hexagonal ZnO nanorods. In agreement with the literature, peak shifts were observed in the diffraction patterns due to metal enrichment. I-V measurements showed a linear relationship between current and voltage across different temperature and voltage ranges. The current of the metal-decorated ZnO nanorods increased with temperature, behavior likely attributed to the semiconducting nature of the samples. At 200 °C, the current values of the metal-decorated ZnO nanorods ranked from highest to lowest as Cr, Ni, Co, and Cu. The electrical analysis results demonstrate that temperature significantly affects the electrical behavior of these nanostructures, with thermally activated conduction mechanisms dominating at higher temperatures. Specifically, Cr-, Ni-, and Cu-decorated ZnO nanorods exhibited linear temperature-dependent current behavior, suggesting thermal activation, while Co-decorated ZnO nanorods were better described by the variable range hopping (VRH) model. The calculated activation energies and coefficient of determination (R^2^) values further supported these distinctions, emphasizing the unique conduction mechanisms across different metal decorations. The gas-sensing experiments revealed that the type of metal decoration on ZnO nanorods critically influences their sensitivity and selectivity toward various gases. Ni- and Co-decorated ZnO nanorods exhibited high sensitivity to hydrogen gas, while variations in current due to ethanol and chloroform exposure highlighted the potential for selective gas detection. The observed behavior can be attributed to electronic and chemical sensitization mechanisms, with metal nanoparticles altering surface barrier layers and modulating charge carrier concentrations.

The study underscores the importance of surface modification in tailoring the electronic and sensing properties of ZnO nanorods for sensor applications. Transition metal doping, as evidenced by enhanced oxygen vacancies and defect-related adsorption sites, plays a pivotal role in optimizing gas-sensing performance. The findings align with previous studies, confirming the potential of metal-decorated ZnO nanorods as highly responsive and selective materials for gas sensing. Future work could focus on exploring the long-term stability and scalability of these nanostructures in practical sensing devices.

## Figures and Tables

**Table 1:** The calculated activation energies of all metal decorated ZnO nanorods.

Materials	Activation Energy (eV)	
	E_A1_	E_A2_
Ni-ZnO Nanorods	0.2	0.63
Cu-ZnO Nanorods	0.16	0.35
Cr-ZnO Nanorods	0.42	
Co-ZnO Nanorods	0.22	

**Table 2:** Comparison of metal oxide-based gas sensors functionalized with noble and non-noble metal nanoparticles in different morphologies.

Sensitive Layer	Target Gas	Test Concentration (ppm)	Sensor Response	Operation Temperature (°C)	REF
ZnO NRs/Pt NPs	H_2_S	0.1	23.1	260	[22]
ZnO NRs/Pd NPs	CH_4_	1000	0.368	80	[64]
ZnO NWs/Au NPs	C_2_H_5_OH	100	33.6 ~5.2	380 200	[65]
ZnO NS/Ag NPs	C_2_H_5_OH	100	28.78 ~5.5	270 210	[66]
ZnO TF/Ir ZnO TF/Ru ZnO TF/IrRu	C_2_H_5_OH	80	13.22 9.63 3.99	250 250 250	[67]
rGO-WO3 NPs/Ni NPs	C_3_H_6_O	3500	6.2	246	[68]
ZnO NS/Co NPs	2-Butanone C_2_H_5_OH	100 100	2540 600	270 300	[69]
ZnO NRs/Ni NPs	H_2_	5000	14.83	200	In this Study
ZnO NRs/Cr NPs	C_2_H_5_OH	5000	3.35	200	In this Study

## Data Availability

The data presented in this study are available on request from the corresponding author.
